# Obesity and age-related alterations in the gene expression of zinc-transporter proteins in the human brain

**DOI:** 10.1038/tp.2016.83

**Published:** 2016-06-14

**Authors:** R H Olesen, T M Hyde, J E Kleinman, K Smidt, J Rungby, A Larsen

**Affiliations:** 1 Department of Biomedicine, Aarhus University, Aarhus, Denmark; 2 Lieber Institute for Brain Development, Johns Hopkins Medical Campus, Baltimore, MD, USA; 3 Department of Neurology, Johns Hopkins University School of Medicine, Baltimore, MD, USA; 4 Department of Psychiatry and Behavioral Sciences, Johns Hopkins University School of Medicine, Baltimore, MD, USA; 5 Center for Diabetes Research, Gentofte University Hospital, Hellerup, Denmark

## Abstract

The incidence of Alzheimer's disease (AD) is increasing. Major risk factors for AD are advancing age and diabetes. Lately, obesity has been associated with an increased risk of dementia. Obese and diabetic individuals are prone to decreased circulating levels of zinc, reducing the amount of zinc available for crucial intracellular processes. In the brain, zinc co-localizes with glutamate in synaptic vesicles, and modulates NMDA receptor activity. Intracellular zinc is involved in apoptosis and fluctuations in cytoplasmic Zn^2+^ affect modulation of intracellular signaling. The *ZNT* and *ZIP* proteins participate in intracellular zinc homeostasis. Altered expression of zinc-regulatory proteins has been described in AD patients. Using microarray data from human frontal cortex (BrainCloud), this study investigates expression of the SCLA30A (*ZNT*) and SCLA39A (*ZIP*) families of genes in a Caucasian and African-American sample of 145 neurologically and psychiatrically normal individuals. Expression of *ZNT3* and *ZNT4* were significantly reduced with increasing age, whereas expression of *ZIP1, ZIP9* and *ZIP13* were significantly increased. Increasing body mass index (BMI) correlated with a significant reduction in *ZNT1* expression similar to what is seen in the early stages of AD. Increasing BMI also correlated with reduced expression of *ZNT6*. In conclusion, we found that the expression of genes that regulate intracellular zinc homeostasis in the human frontal cortex is altered with increasing age and affected by increasing BMI. With the increasing rates of obesity throughout the world, these findings warrant continuous scrutiny of the long-term consequences of obesity on brain function and the development of neurodegenerative diseases.

## Introduction

Worldwide, populations are growing older.^1^ A downside to increased longevity is the growing number of dementia cases, most commonly in the form of Alzheimer's disease (AD).^2^ Today, 36 million people suffer from AD, with the expectation that this number will double within just 20 years^3^ implosing a huge socioeconomic burden on societies.

Most cases of AD are sporadic, occurring late in life. The main risk factors of sporadic AD are advanced age ApoE genotype and diabetes.^4, 5^ Moreover, several studies find that obesity *per se* increases the risk of developing dementia.^6, 7, 8, 9, 10, 11^ There is a correlation between obesity in early and midlife and the subsequent development of AD in later years. Interestingly, obesity in the elderly might, in fact, be protective.^7^ The link between neurodegeneration and obesity should attract concern from the health-care communities as there is a dramatic world-wide increase in average body weight in recent decades.^12, 13^

The mechanisms linking metabolic dysfunction and obesity to neurodegenerative processes are unclear. A high incidence of cardiovascular disease, persistent low-grade inflammation and low levels of zinc in the blood are common in diabetic and obese individuals.^14, 15, 16, 17, 18^ Both circulating and erythrocyte zinc levels are reduced^16^ in obesity and there appears to be a direct link between zinc deficiency and obesity-related comorbidities such as insulin resistance.^15, 17, 18^ Inflammation and atherosclerosis likely explain part of the epidemiological linkage between dementia, metabolic disease and obesity.^19, 20^ The increased risk of AD in diabetic individuals, independent of body weight, is a subject of great interest.

As the human body does not sequester stores of zinc for later use, plasma zinc becomes the main pool of readily available zinc. Consequently, hypozincemia potentially could affect the zinc homeostasis throughout the body. The relationship between brain zinc levels and obesity are unknown. In AD brains, the literature is inconclusive and varies dependent on the anatomical localization of the sample. The areas rich in zinc include hippocampus and neocortex, especially layers III and IV, in which the so-called zinc-enriched neurons are found.^21^ Moreover, zinc binds to amyloid plaques^22^ and there have been reports of both high^23, 24^ and low^25, 26^ zinc content in AD brains compared with healthy brains.^27^

The functional relationships between zinc homeostasis, the amount of zinc present in the human brain and the onset of neurodegenerative processes require additional inquiry. In zinc-enriched neurons, the amount of free synaptically active zinc is controlled by the *ZNT3* transporter.^28^ The *ZNT3* downregulation has been linked both to AD pathology and to normal aging of the brain.^29, 30^ Within synaptic vesicles, free zinc co-localizes with glutamate, and experimental studies indicate that the liberation of zinc from zinc-enriched neurons can modulate NMDA activity.^31^ The ZnT3-knockout mice exhibit cognitive difficulties with increasing age^30^ and moderate dietary zinc deficiency has been linked to cognitive deficits in humans.^32^ The role of vesicular zinc in amyloid pathology has been tested by cross-breeding the Tg2576 AD mice with ZnT3KO mice.^33^ This experiment confirmed a role for vesicular zinc in plaque formation as the resulting double-mutant mice displayed a significantly reduced plaque load. However, the mechanisms linking zinc, plaque formation and cognitive performance remains elusive. Dietary zinc restriction paradoxically leads to increased plaque formation in the APP/PS1 AD mouse.^22^ Similarly, zinc supplementation reduced plaque formation in an AD model.^34^ In that experiment, zinc supplementation also coincided with reduced cognitive performance. Others have shown that alterations in intracellular zinc might also disturb microtubular function and the formation of neurofibrillary tangles.^35^

Overall, most experiments with zinc supplementation seem to improve cognitive performance,^36^ but the diversity in literature on this topic highlights the importance of a tightly balanced zinc regulation.

Alterations in intracellular zinc homeostasis may be produced by alterations in the *ZNT* or *ZIP* proteins. The *ZIP* proteins regulate zinc uptake from the extracellular environment as well as zinc release from intracellular stores. In contrast, the *ZNT* proteins re-direct zinc to the extracellular space and provide zinc for synaptic vesicles (*ZNT3*) and other organelles.

Altered gene or protein expression of several ZNTs has been associated with AD pathology. Although *ZNT3* expression is downregulated, other *ZNT* transporters are upregulated. In hippocampus, there are reports of increased protein levels of *ZNT4*, *ZNT6* and *ZNT1*.^37, 38^ The *ZNT1* transporter is the only member of the SCLC30A family actively transporting zinc from the cytoplasm to the extracellular compartment. Post-mortem analyses have shown that *ZNT1* expression is reduced in AD as well as in patients suffering from mild cognitive impairment.^38^ On the other hand, patients with fulminant AD displayed an increased expression of *ZNT1*.^38^

On the basis of previous studies, we hypothesize that a reduced level of *ZNT1* in early-stage dementia constitutes a possible defense mechanism against systemic or local zinc deficiencies.

Utilizing cDNA microarray data from the prefrontal cortex of 145 neurologically and psychiatrically sound individuals, the relationship between body mass index (BMI), a standard measurement of weight/height relation and the *ZIP* and *ZNT* gene expression was examined. Furthermore, we investigated the relationship between the expression of these genes in the frontal cortex, gender or race, comparing African-American to Caucasian cohorts.

## Materials and methods

The data used in this study were obtained through the BrainCloud database (http://braincloud.jhmi.edu/) courtesy of the Lieber Institute of Brain Development. We used microarray data on post-mortem dorsolateral prefrontal cortex (corresponding to Broadman's areas number 9 and 46) from individuals without any psychiatric or neurological disease. The microarray expression data were obtained using the Illumina Oligoset (HEEBO7) chip, processed and expressed as previously described.^39^ All tissue collection was performed with informed consent obtained from the next of kin. All the data were subsequently anonymized in accordance with the rules and regulations of the National Institute of Health (using protocol 90-M-0142).

### BrainCloud demographics

BrainCloud contains non-neurologic/non-psychiatric controls up to 78 years of age. None of the subjects had any pathological findings consistent with AD, or other neurological diseases. In the present study, we excluded all individuals under the age of 18, four of Asian descent and five of Hispanic descent. Focusing on obesity *per se*, four individuals with known diabetes and three individuals with unknown BMI were excluded leaving 145 (63 Caucasians and 85 African Americans) subjects for the study. For a detailed description of the demographics of these subjects, please see Table 1.

### Genes

All genes of the SLC30A/39A families available in BrainCloud were analyzed in this study. Accordingly, all members of the SLC30A family (*ZNT1*–*ZNT10*) were analyzed, as well as most members of the SCL39A family with the exception of the *ZIP5, ZIP6* and *ZIP1*2 genes that are not included on the Illumina HEEBO chip.

### Statistics

Statistical analyses were done using R version 3.0.2, (R Foundation for Statistical Computing; http://www.r-project.org/). The data were considered normally distributed from examination of the quantile–quantile plot (QQ-plot), linear regression analysis was performed to assess the contributions of age on mRNA expression levels. The variation in mRNA expression of each gene was analyzed using BMI and age as continuous variables. The interaction between the age and BMI for each gene was compared using multiple regression analysis (Table 2). The comparison between African Americans and Caucasians, males and females, were performed using Student's *t*-test as when data were normally distributed, when not normal distributed Wilcoxon rank-sum test was used. Student's *t*-test is written as *t*(degrees of freedom)=, *P*=. Wilcoxon rank-sum is written as W=rank sum, *P*=0. For all analyses, level of statistical significance was set at *P*⩽0.05.

## Results

### Increasing BMI results in a decrease in the mRNA expression of *ZNT1* and *ZNT6*

**Figure 1:**
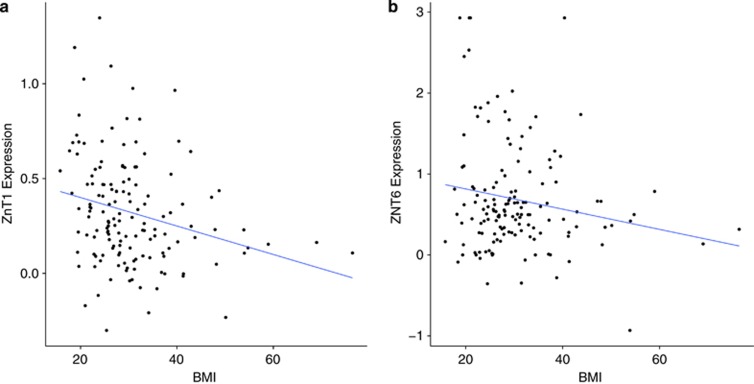
(**a** and **b**) On the *x* axis, BMI (kg/m^2^) and on the *y* axis, there are mRNA expressions of *ZNT1* (*P*=0.002) (**a**), *ZNT6* (*P*=0.03) (**b**). Expression is expressed as log_2_ of the ratio of sample signal to the reference signal Each dot represents one individual. Line represents best-fitted line using regression. BMI, body mass index.

Looking at BMI as a continuous variable revealed a statistically significant inverse correlation between *ZNT1* and *ZNT6*. For *ZNT1*: *ZNT1* expression=−0.007507 × BMI+0.550087, F(1,143)=9.912, *R*^2^=0.065, *P*<0.005. For *ZNT6*: *ZNT6* expression=−0.012496 × BMI+1.065286, F(1,143)=4,681, *R*^2^=0.0319, *P*=0.032. There is an inverse correlation between mRNA expression and increasing BMI (Figures 1a and b). The mRNA expression of the rest of the *SLC30* family, that is, *ZNT2*, *ZNT3*, *ZNT4*, *ZNT5*, *ZNT7*, *ZNT8*, *ZNT9* and *ZNT10* did not correlate with BMI. In the *SLC39A* family, increasing BMI did not correlate with the mRNA expression of the *ZIP*s examined, that is, *ZIP2–4*, *ZIP7–11* and *ZIP13–14*. We found interaction between BMI and age for the genes *ZIP1* and *ZIP11*. In *ZIP1*, we found an interaction between age and BMI, and used this in the regression, giving us a significant relationship between *ZIP1*, age and BMI: *ZIP1*=−0.0108695 × age−0.0213034 × BMI+0.0005721 × (Age × BMI)+0.0451270, F(3,141)=4.98, *R*^2^=0.09581, *P*=0.0026; giving us a combined decrease with age and BMI for ZIP1.

In ZIP11, we did not find any relationship with gene expression and age or BMI; ZIP11=−0.0089901 × Age−0.0149509 × BMI+0.0004191 × (Age × BMI)+0.4999, F(3,141)=2.508, *R*^2^=0.05065, *P*=0.06.

### The mRNA expression of *ZNT3* and *ZNT4* is gradually reduced throughout adulthood, whereas the expression of *ZIP9* and *ZIP13* gradually increases

**Figure 2:**
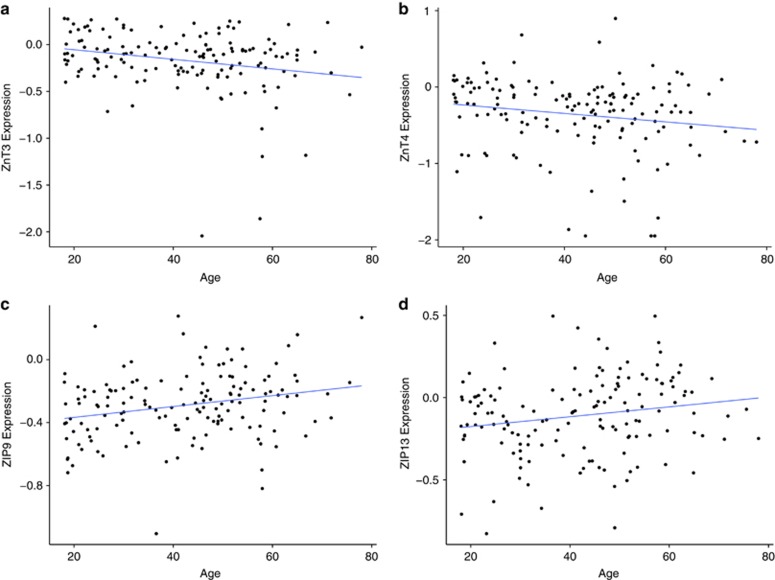
(**a**–**d**) On the *x* axis, Age (years) and on the *y* axis, there are mRNA expressions of *ZNT3* (*P*<0.005) (**a**) and *ZNT4* (*P*=0.038) (**b**), *ZIP9* (*P*=0.0024) (**c**) and *ZIP13* (*P*=0.0226) (**d**). Expression levels are measured as log_2_ of the ratio of sample signal to the reference signal. Each dot represents one individual. Line represents best-fitted line using regression.

Looking at each gene individually, a statistically significant reduction in the mRNA expression of *ZNT3* (Figure 2a; *ZNT3* expression=−0.005144 × Age+0.047144, F(1,146)=8.456, *R*^2^=0.05475, *P*<0.004) and *ZNT4* (Figure 2b; *ZNT4* expression=−0.005535 × Age−0.123892, F(1,146)=4.384, *R*^2^=0.029, *P*=0.038) was seen with increasing age throughout adult life (>18 years), whereas the expression pattern of the remaining eight efflux transporters of the *SLC30A* family was unaffected. In the *SLC39A* family of zinc influx proteins, there was a statistically significant upregulation of *ZIP9* (*ZIP9* expression=0.003454 × Age−0.435929, F(1,146)=9.515, *R*^2^=0.06118, *P*=0.002) and ZIP13 (*ZIP13* expression=0.002995 × Age−0.236005, F(1,146)=5.313, *R*^2^=0.03511, *P*=0.023) with increasing age (Figures 2c and d). Likewise, there were decreased levels of *ZIP10*, (*ZIP10* expression=−0.008570 × Age+0.964997, F(1,146)=4.632, *R*^2^=0.03075, *P*=0.033)

There was no relationship between age and the mRNA expression of *ZIP2*–*4*, *ZIP7*–8 or *ZIP14*.

### Smoking does not affect mRNA expression of zinc-regulatory proteins of the *SLC30A* (*ZNT*) and *SLC39A* (*ZIP*) families

The relationship between smoking status at the time of death on the gene expression of the *SLC30A* (*ZNT*) family and most of the *SLC39A* family, that is, *ZIP1*–*4, ZIP7*–*11* and *ZIP13*–*14* was examined without any significant findings on the mRNA expression of either class of zinc-regulatory proteins.

### Analyses of biological variations in the mRNA expression of zinc-regulatory proteins revealed both sex differences and differences between African Americans and Caucasians

**Figure 3:**
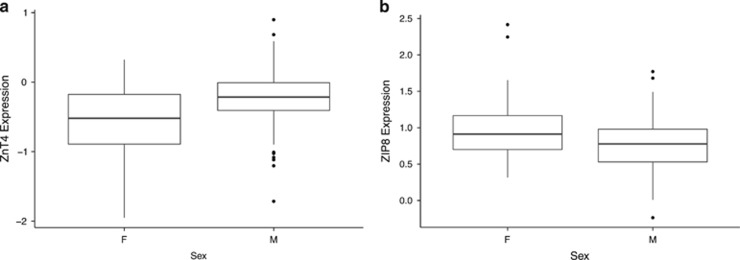
(**a** and **b**) On the *y* axis, mRNA expression of ZnT4 (**a**) and ZIP8 (**b**). F, female; M, male. For each group, bold line represents median; box is upper and lower quantile; and whiskers are upper and lower extreme. Expression levels are measured as log_2_ of the ratio of sample signal to the reference signal.

**Figure 4:**
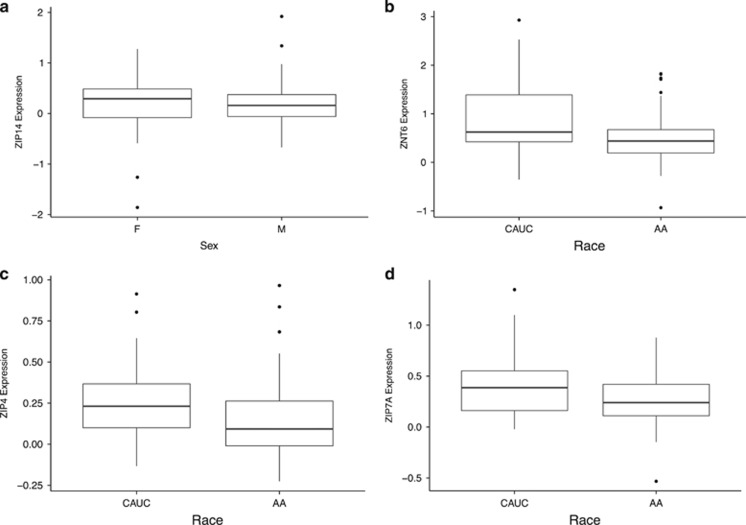
(**a**-**d**) On the *y* axis, mRNA expression, *ZIP14* (**a**), *ZNT6* (**b**), *ZIP4* (**c**) and *ZIP7A* (**d**). CAUC, Caucasian; AA, African American. For each group, bold line represents median; box is upper and lower quantile; and whiskers are upper and lower extreme. Expression levels are measured as log_2_ of the ratio of sample signal to the reference signal.

We found that men exhibited a statistically, significantly higher expression of *ZNT4* (*t*(62.446)=−3.7279, *P*<0.0004; Figure 3a) than women in this study as well as statistically, significantly lower expression of *ZIP1* (*t*(65.14)=2.9317, *P*=0.01241) and *ZIP8* (*t*(68.837)=−2.9317, *P*=0.009; Figure 3b). Comparing the gene expression pattern of zinc-regulatory proteins between racial groups, we found that African Americans displayed a statistically, significantly higher expression of *ZIP14* (*t*(137.882)=−3.4778, *P*=0.0007; Figure 4a), as well as a statistically significant lower expression of three members of the *SLC30A* family, that is, *ZNT6* (*t*(92.96)=3.7504, *P*=0.003; Figure 4b), and two of the *SLC39A* genes, that is, *ZIP4* (*W*=3511, *P*=0.001) and ZIP7 (*t*(113.358)=3.1329, *P*=0.0022; Figures 4c and d).

## Discussion

The analysis of the mRNA expression of the entire *SLC30A* family as well as 11 of the 14 *SLC39A* genes in the present study provides new insight into the intracellular zinc regulation in the human brain. Our analyses confirm a previous study^30^ that *ZNT3* downregulation is indeed a feature of human aging, accompanied by the novel finding of a downregulation of *ZNT4* and upregulation of *ZIP1, ZIP9* and *ZIP13*. Furthermore, we identified an association between increased body weight and altered zinc homeostasis in the brain as increasing BMI correlated with statistically significant decreases in the expression of *ZNT1 and ZNT6*. Interestingly, each of these zinc transporters previously has been linked to the development of AD.^29, 30, 37, 38, 40^

Low plasma zinc in association with obesity is well described.^14, 15, 16, 17, 18^ There is scant information about the zinc content of the human brain in relationship to obesity. Genetically obese mice show unaltered or slightly reduced zinc concentrations in the brain.^41^ Previous reports on the ob/ob mice also found that, compared with lean mice, these obese mice had an increased intestinal uptake of zinc. Such increased zinc uptake occurred irrespective of the amount of food ingested, indicating that in this form of obesity, there is an altered intestinal absorption of zinc.^41, 42^ It is unclear whether this increased uptake of zinc has a causative role in this form of obesity, or is an epiphenomenon. A later study by Chen *et al.*^43^ did, however, find that zinc supplementation significantly increased body fat accumulation in both the ob/ob mice and the obese ICR mice. These findings suggest that increased uptake of zinc could have a causative role in obesity. Paradoxically, the obese mice displayed reduced zinc content in most of the body—that is, the skin, muscle and bone—despite the increased zinc uptake. On the other hand, the amount of zinc in liver, adipose tissue and intestines increased in the obese animals, indicating that obesity alters the zinc distribution in the body.^41, 44^ In this respect, zinc sequestration in the enlarged liver could have a prominent role.^44^

Plasma zinc constitutes the main readily available source in the body. Hence, obesity-induced hypozincemia will likely result in reduced zinc availability for the brain. Several studies^14, 15, 16^ describe a reduced level of circulating zinc in obese individuals from which affect both total zinc levels and mean erythrocyte zinc levels. In morbidly obese eligible for bariatric surgery, as many as 73.9% were zinc deficient.^14^ Zinc deficiency correlates with elements of the metabolic syndrome such as insulin secretion,^15, 17, 18^ as well as with diabetes as such in which an increased loss of zinc in the urine is also seen.^18^ In general, an increased plasma volume could contribute to this pattern, as could reduce dietary zinc intake, which have been reported among obese individuals.^17^ As recently reviewed by Takeda *et al.*,^45^ dietary zinc deficiency rarely results in measurable reductions in brain zinc concentrations. Physiologically, on the other hand, the sensitivity of the body to zinc deficiency is reflected by impaired learning and increased production of glucocorticosteroids as seen in several animal studies.^32, 46, 47, 48^ As described by Stanstead,^32^ several neuropsychological studies indicate that marginal zinc deficiency might affect perception, attention or psychomotor skills in humans and that this can be corrected by zinc alone or in combination with other micronutrients. In addition, a recent study found that zinc supplementation reduced symptoms of depression as registered by the Hamilton Depression Rating Scale and increased plasma-BDNF levels in obese and overweight depressed humans.^49^ Similarly, several studies link zinc supplementation to increased cortical, cerebral and hippocampal levels of BDNF in mice.^50, 51, 52^

Obesity in midlife is associated with an increased risk of AD.^6, 7, 8, 9, 10, 11^ In some asymptomatic individuals in their late 40s and early 50s, neuropathological examination has discerned early signs of AD pathology^53^ suggesting that the pathological changes associated with AD may begin far earlier than commonly anticipated. Recent works of Lowell *et al.*^38^ have studied alterations in intracellular zinc regulation in relation to the progression from mild cognitive impairment to fulminant AD. Although some variation with anatomical location within the brain was seen, this study indicated that *ZNT1* protein expression is increased in the pre-clinical and early stages of AD, and subsequently decreases in the late stages.^38^ These findings suggest that dysregulation of intracellular zinc signaling participates in AD development, but in a complex fashion. In addition, a recent study has shown that *ZNT1* binds directly to the GluN2A subunit of the NMDA receptor,^54^ suggesting that alterations in the expression of this specific zinc transporter may have functional consequences not directly related to zinc *per se*. The exact role of *ZNT1* in synaptic activity remains to be elucidated, but this *ZNT1*–GluN2A complex formation appears to be dynamic in nature as the tendency towards such complex formation varies with synaptic activity.

In addition to alterations in *ZNT1* expression, *ZNT4* and *ZNT6* protein levels are upregulated in late AD.^37^ The *ZNT4* and *ZNT6* proteins transport zinc into the lysosomal and trans-golgi compartments and it has been hypothesized that an upregulation of *ZNT4* and *ZNT6* would allow excess zinc to be removed from the cytoplasm before doing any harm.^55^ On the other hand, upregulation of *ZNT4* and *ZNT6* might also reflect the relative increase in glial cells in late AD. In this study, we found that *ZNT5* and *ZNT6* are significantly downregulated with increasing BMI, suggesting that obesity could potentially affect tightly regulated zinc homeostasis in the brain tissue.

Investigating zinc dyshomeostasis in AD pathology, both increased and reduced levels of cytoplasmatic zinc have been implicated. Intracellular zinc depletion destabilizes microtubules, herby starting a cascade of tau release, hyper-phophorylation and formation of neurofibrillary tangles.^34^ Intracellular zinc excess, occurring as a consequence of Amyloid beta aggregation and reactive oxygen species generation, liberates zinc from metallothionein and may affect mitochondrial function and induce apoptosis.^56, 57, 58, 59^ One way to reconcile these seemingly contradictory findings is to suggest that intracelluar zinc must be tightly regulated to avoid adverse molecular consequences.

Other studies have implicated zinc transporters *ZNT3* and *ZNT10* in AD.^29, 30, 40^ Here we find that their expression varies with age and BMI, respectively. An age-related *ZNT3* downregulation has been reported in an older human population (*n*=24)—age 49–91 years—as well as in aging rodents.^30^ The *ZNT3* protein is responsible for the accumulation of zinc in synaptic vesicles and could be involved in memory and learning.^28^ A reduction in *ZNT3* expression has been found in the brains of AD patients. Moreover, the role of *ZNT3* in learning and memory is suggested by age-related cognitive loss in elderly *Znt3* knockout mice.^30^ The investigation of *Znt3* gene and protein expression in the SAMP10 age-accelerated mice also found a link between aging, *ZNT3* downregulation and reduced zinc content in the brain.^60^ *ZNT3* gene downregulation increases senescence in vascular smooth muscle cells and interestingly, *ZNT10* has the same effect.^61^ *ZNT10* was recently identified in brain tissue and a decrease in *ZNT10* expression has been seen in AD brains.^40^ Despite this close relationship between *ZNT3* and *ZNT10*, we do not see an age-related decrease in *ZNT10* expression in our study population.

The role of the *SLC39A* family in aging has not previously been investigated. Here we find that three members of the *ZIP* family, that is, *ZIP1, ZIP9* and *ZIP13* are upregulated with increasing age. Of these, *ZIP1* is believed to be ubiquitously expressed in the body.^62^ *ZIP1* is highly expressed in the hippocampus and in murine studies, and knocking out *ZIP1* attenuated seizure-induced neuronal death.^63^ *ZIP1* expression can be upregulated by interleukin-6 and at least in the prostate also is increased by testosterone.^62^ Dietary zinc deficiency mainly affects the subcellular location of *ZIP1* leading to an increased presence in the plasma membrane,^64^ highlighting the need for further studies of the protein levels of *ZIP1* in the aging brain. Little is known of the function of *ZIP9* and *ZIP13* in the brain. Recent studies do, however, link *ZIP9*-mediated zinc signaling to Akt/Erk regulation^65^ and steroid receptor^66^ function in the other tissues. *Zip 13* has previously been described as important in human retino-pigmental cells,^67^ although this transporter may, in fact, be important for the transport of other trace metals such as iron as well.^68^

## Conclusion

The present study shows that both increasing age and increasing BMI are associated with alterations in the expression of genes mediating intracellular zinc homeostasis in the human brain. Individual members of the *ZNT1* and *ZIP* gene families have differing relationships with increasing age, as mRNA expression of ZNT3 and ZNT4 is downregulated, whereas the expression of *ZIP3*, *ZIP9* and *ZIP13* increases. Increasing BMI levels are associated with reduced expression of *ZNT1* and *ZNT6*, which previously have all been implicated in AD pathology. In light of the increasing rates of obesity and AD throughout the world, more studies on the physiological consequences of zinc dyshomeostasis and the combined role of *ZIP* and *ZNT* proteins are warranted. In this respect, modern techniques such as next-generation sequencing could give us further knowledge on the role of zinc transporters in AD and obesity.

## Figures and Tables

**Table 1:** Demographics of the adult (>18 years of age) subjects of African-American and Caucasian descent obtained from the online BrainCloud data set.

*BMI group*	n	*Race*	*Sex*	*Age*	*BMI*
Underweight (<18.5)	4	1 CAUC/3 AA	1 F/3 M	38.58 (20.2)	17.52 (1.2)
Normal (18.5–24.99)	39	20 CAUC/19 AA	17 F/22 M	39.91 (17.6)	22.2 (1.9)
Overweight (25–30)	41	21 CAUC/20 AA	5 F/36 M	44.2 (14.1)	27.42 (1.35)
Obese (>30)	61	20 CAUC/41 AA	23 F/38 M	41.79 (13.9)	38.61 (9.5)
NA	3	NA	NA	NA	NA
Total	148	63 CAUC/85 AA	46 F/102 M	42.1 (15.1)	30.4 (9.78)

Abbreviations: AA, African American; BMI, body mass index; CAUC, Caucasian; F, female; M, male; NA, not available. Results are given as mean (s.d.).

**Table 2:** Interaction between age and body mass index, in relation to analysis of each zinc-transporter gene

*Gene name*	P-*value*
*ZNT1*	0.46
*ZNT2*	0.732
*ZNT3*	0.48
*ZNT4*	0.07
*ZNT5*	0.49
*ZNT6*	0.772
*ZNT7*	0.34
*ZNT8*	0.837
*ZNT9*	0.2
*ZNT10*	0.7
*ZIP1*	0.0143^a^
*ZIP2*	0.0728
*ZIP3*	0.202
*ZIP4*	0.689
*ZIP6*	0.366
*ZIP7*	0.872
*ZIP8*	0.679
*ZIP9*	0.656
*ZIP10*	0.116
*ZIP11*	0.0451^a^
*ZIP13*	0.648
*ZIP14*	0.315

a Significant interaction. Evaluated using multiple regression.
